# Supplementary material for: Apoptosis of intestinal epithelial cells restricts Clostridium difficile infection in a model of pseudomembranous colitis
Source: Nat Commun. 2018 Nov 19;9:4846. doi: 10.1038/s41467-018-07386-5 (PMC6242954; doi:10.1038/s41467-018-07386-5)
Supplement: Supplementary file 1 — Supplementary Information [file 41467_2018_7386_MOESM1_ESM.pdf]

Supplementary Information for:

Apoptosis of intestinal epithelial cells restricts *Clostridium difficile* infection in a model of pseudomembranous colitis

Saavedra et al.

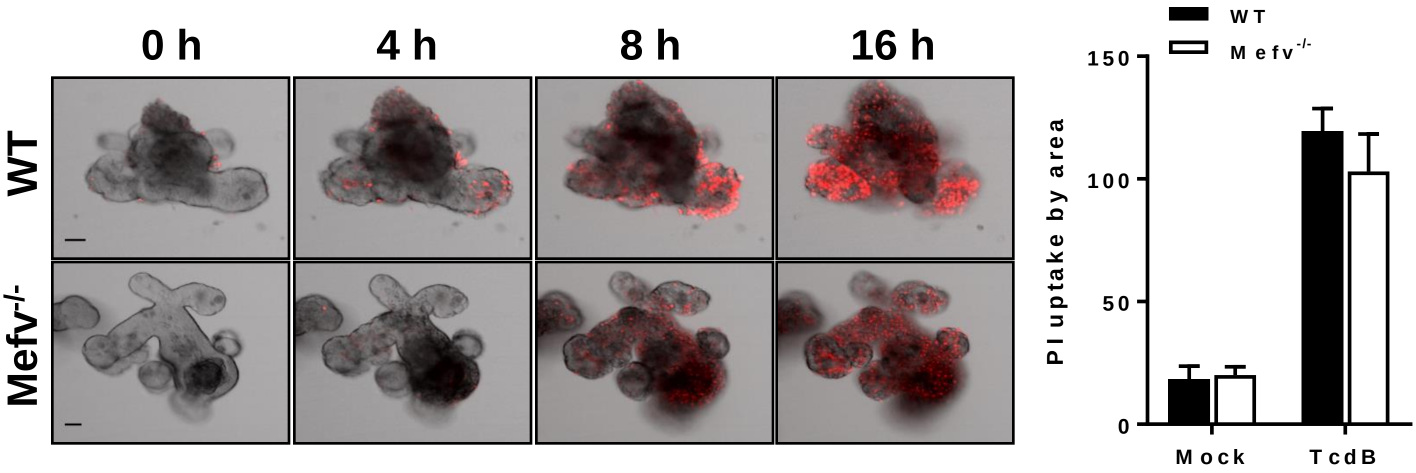

Supplementary Figure 1. Pyrin inflammasome activation and pyroptosis are dispensable for TcdB-induced IEC cytotoxicity. Primary intestinal organoids from wildtype (WT) and *Mefv*$^{-/-}$ mice were stimulated with TcdB before PI incorporation was analyzed by live-imaging for 16 h. Graphs correspond to PI quantification plotted by organoid area. Scale bars: 30 µm. Data are representative of at least 3 independent experiments.

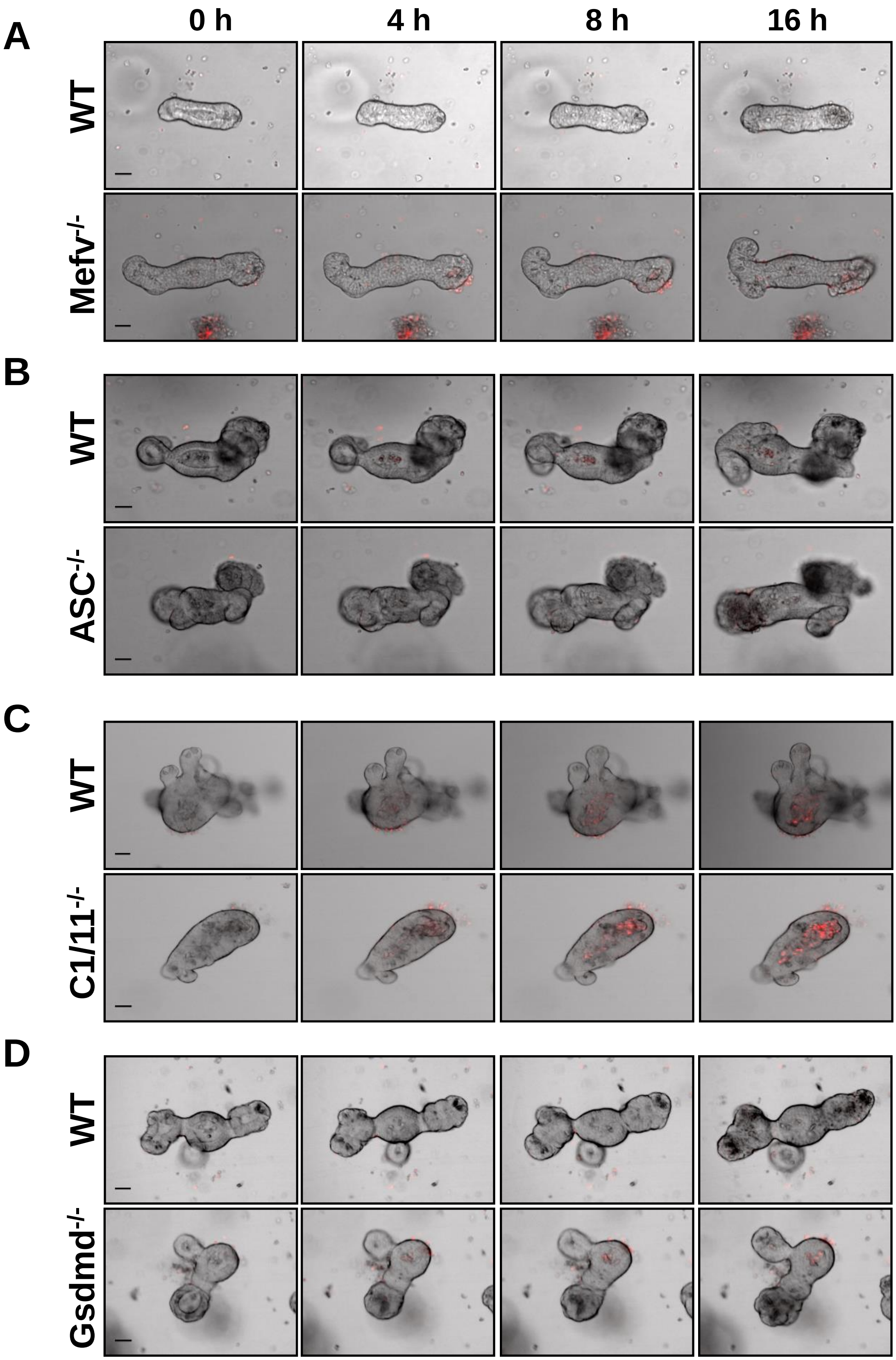

Supplementary Figure 2. Imaging control of *Mefv$^{-/-}$*, *Asc$^{-/-}$*, *Casp1/11$^{-/-}$* and *Gsdmd$^{-/-}$* organoids. A-D, Unstimulated primary intestinal organoids from (A) *Mefv$^{-/-}$*, (B) *Asc$^{-/-}$*, (C) *Casp1/11$^{-/-}$* or (D) *Gsdmd$^{-/-}$* mice analyzed by live-imaging for 16 h. Scale bars: 30 µm. Data are representative of at least 3 independent experiments.

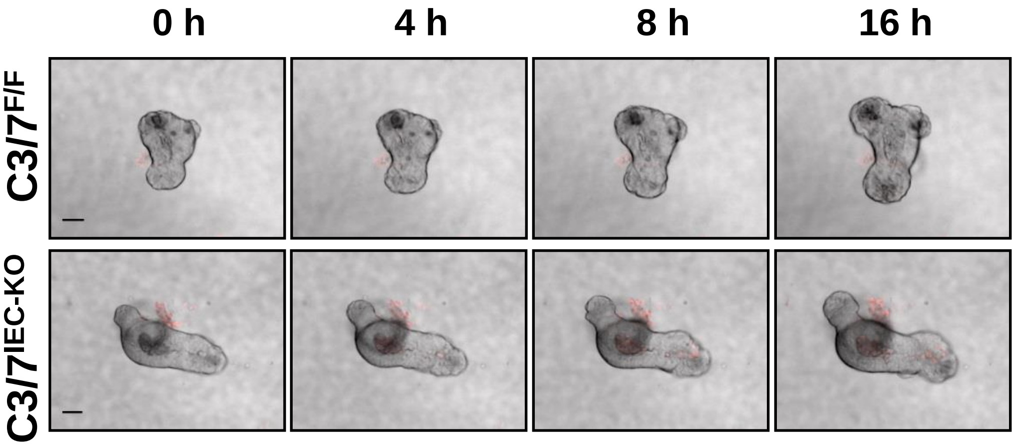

Supplementary Figure 3. Imaging of control *Casp3/7^F/F^* and *Casp3/7^IEC-KO^* IEC organoids. Unstimulated primary intestinal organoids from *Casp3/7$^{F/F}$* and *Casp3/7$^{IEC\text{-}KO}$* mice were analyzed by live-imaging for 16 h. Scale bars: 30 µm. Data are representative of 3 independent experiments.

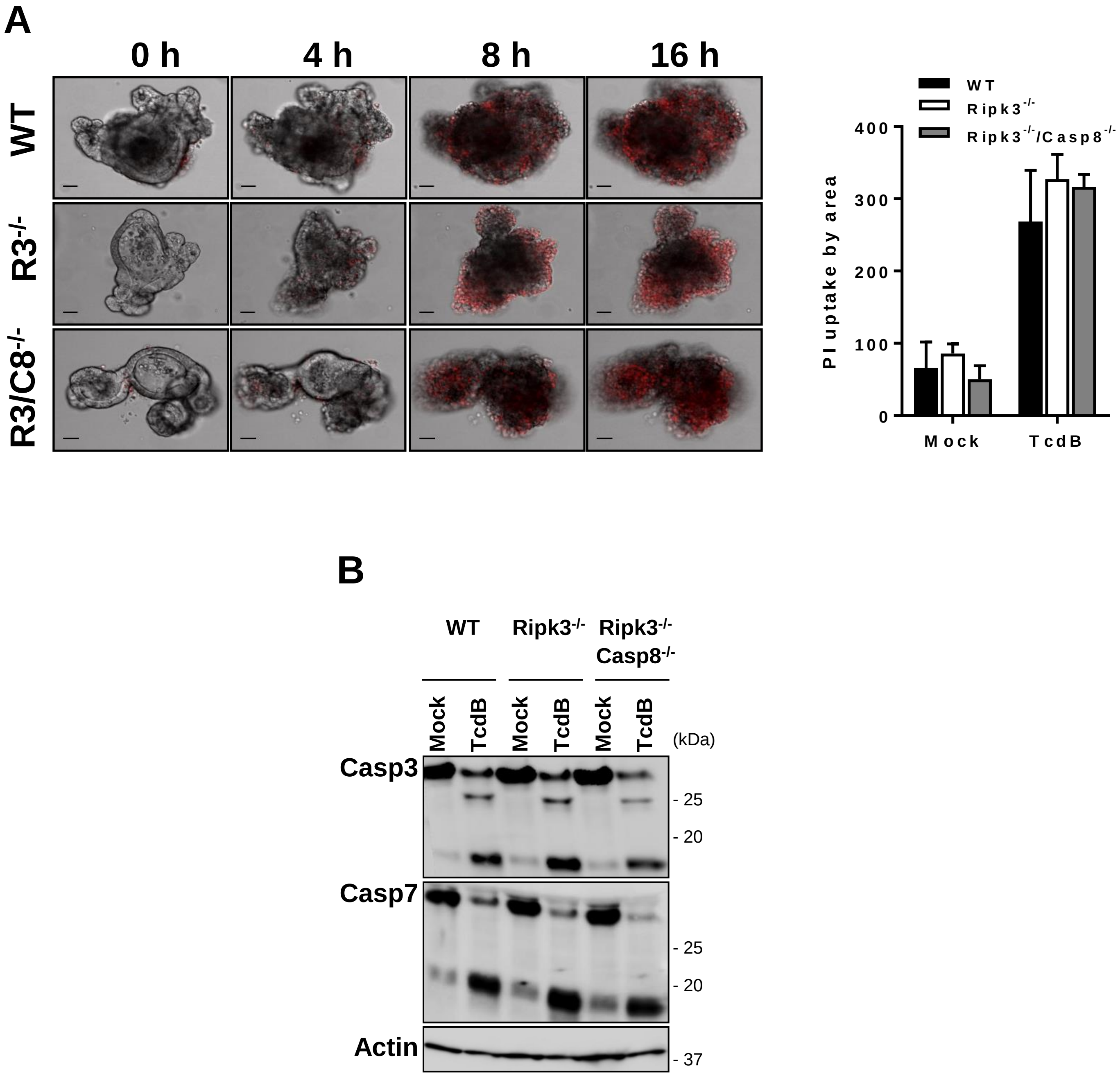

Supplementary Figure 4. RIP kinases and caspase-8 are dispensable for TcdB-induced IEC killing. (**A**) Primary intestinal organoids from wildtype (WT), *Ripk3*$^{-/-}$ and *Ripk3*$^{-/-}$*Casp8*$^{-/-}$ mice were stimulated with TcdB before PI incorporation was analyzed by live-imaging for 16 h. Graphs depict PI quantification plotted by organoid area. Scale bars: 30 µm. (**B**) Cell lysates from primary intestinal organoids of *Ripk3*$^{-/-}$ and *Ripk3*$^{-/-}$*Casp8*$^{-/-}$ or TcdB were immunoblotted for the indicated proteins. Data are representative of 3 independent experiments.

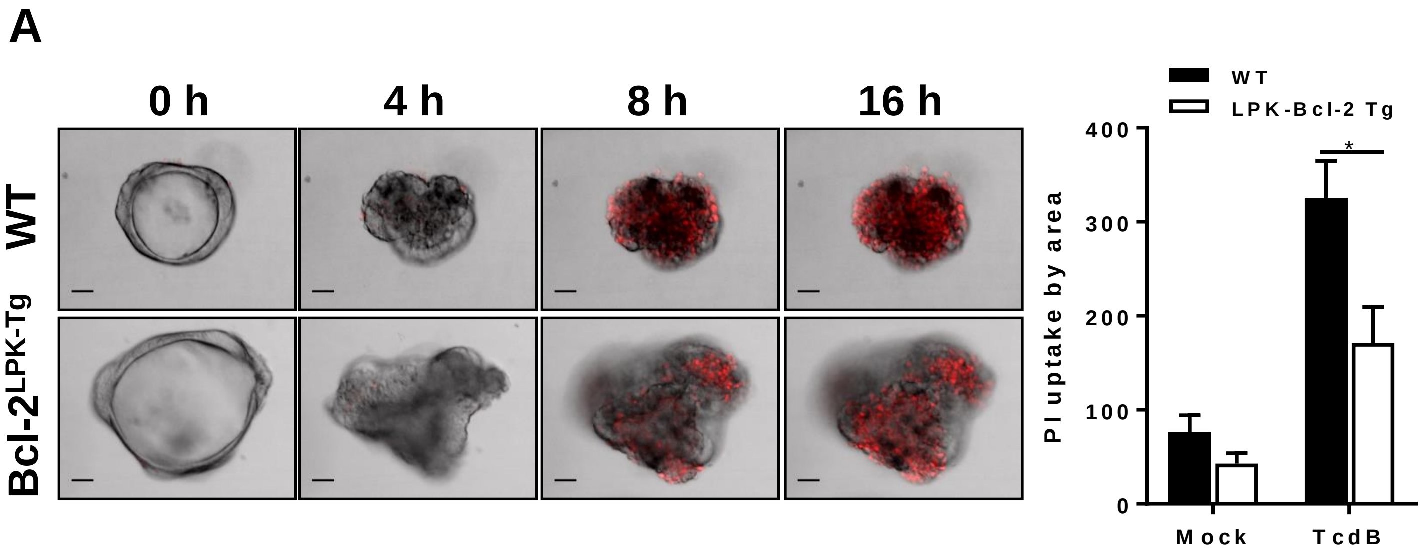

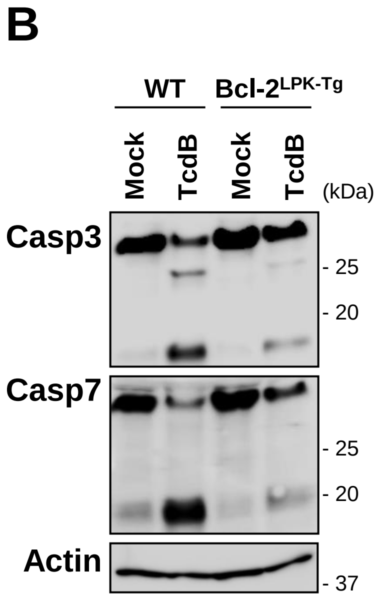

Supplementary Figure 5. Bax/Bak pores contribute to TcdB-induced IEC killing. (**A**) Primary intestinal organoids from wildtype (WT) and *Bcl-2^{LPK-Tg}* mice were stimulated with TcdB before PI incorporation was analyzed by live-imaging for 16 h. Graphs depict PI quantification plotted by organoid area. Scale bars: 30 µm. (**B**) Cell lysates from primary intestinal organoids of *Bcl-2^{LPK-Tg}* stimulated with TcdB were immunoblotted for the indicated proteins. Data are representative of 3 independent experiments. Data are shown as mean ± SD and were analyzed with 2-way ANOVA. *P < 0.05, **P < 0.01, and ***P < 0.001.

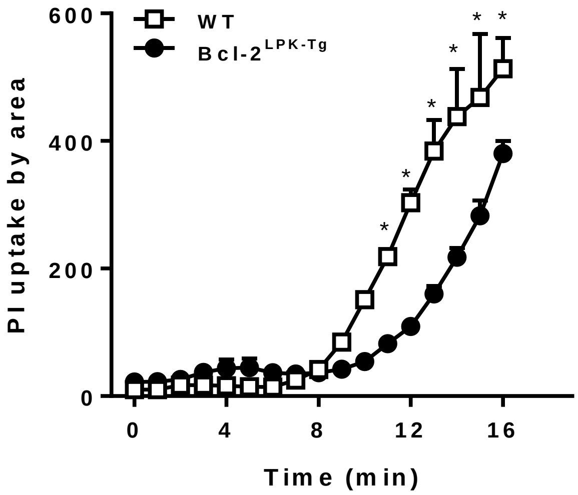

Supplementary Figure 6. RIP kinases and caspase-8 are dispensable, whereas Bax/Bak pores contribute to TcdA-induced IEC killing. Primary intestinal organoids from wildtype and *Bcl-2*$^{LPK\text{-}Tg}$ mice were stimulated with TcdA before PI incorporation was analyzed by live-imaging for 16 h. Graphs depict PI quantification plotted by organoid area in intervals of 1 h. Data are representative of 3 independent experiments. Data are shown as mean ± SD and were analyzed with 2-way ANOVA. *P < 0.05, **P < 0.01, and ***P < 0.001.

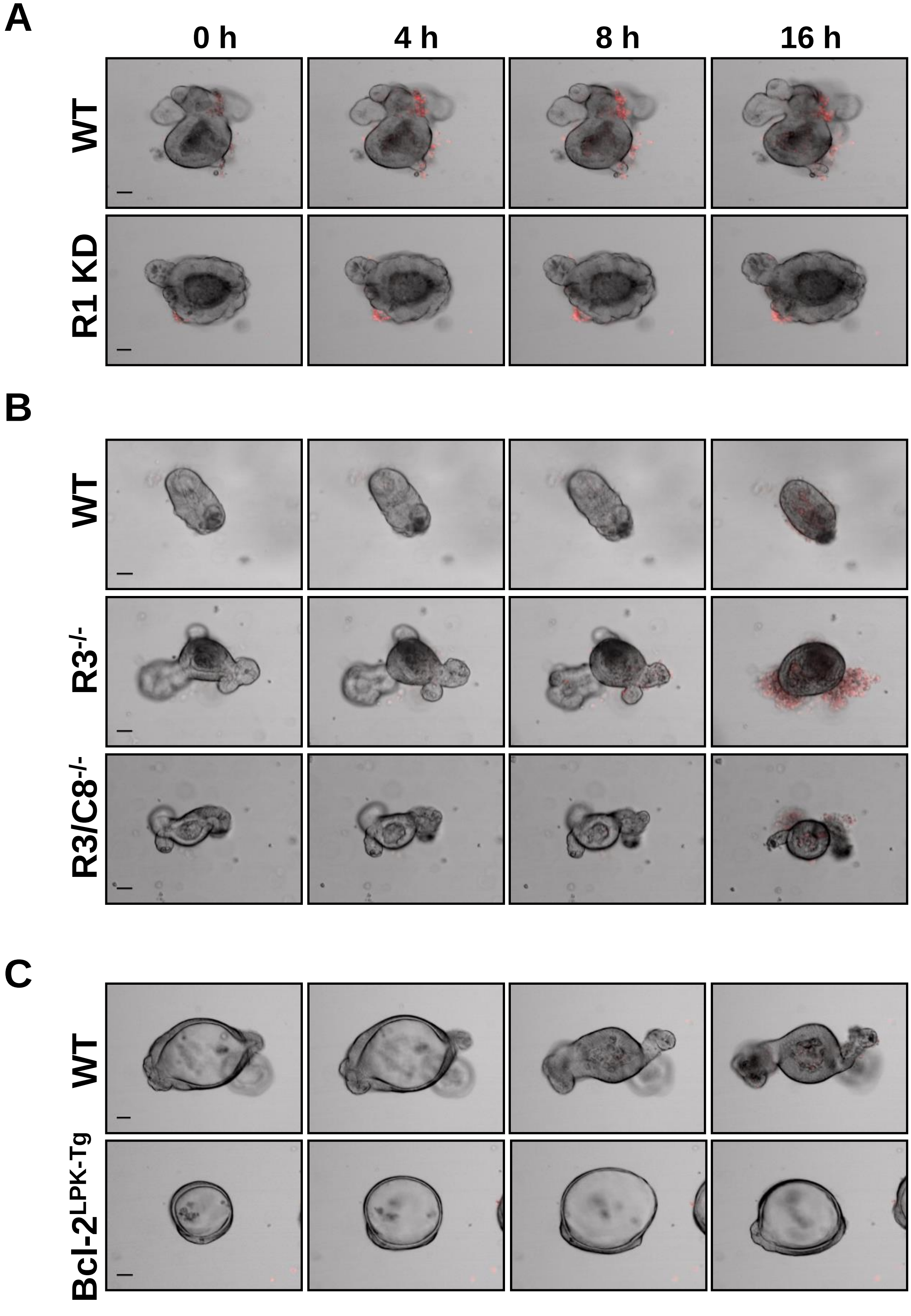

Supplementary Figure 7. Imaging control of IEC organoids. A-C, Unstimulated primary intestinal organoids from (A) *Ripk1*^{D138N} (Ripk1 kinase dead), (B) *Ripk3*$^{-/-}$ and *Ripk3*$^{-/-}$*Casp8*$^{-/-}$ or (C) *Bcl-2*$^{LPK\text{-}Tg}$ mice were analyzed by live-imaging for 16 h. Scale bars: 30 µm. Data are representative of 3 independent experiments.

Fig. 2C

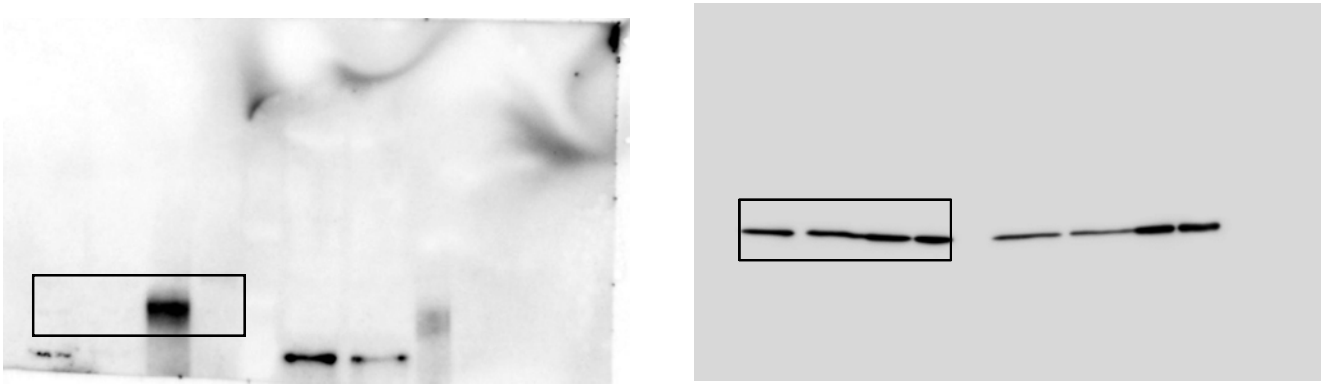

Pyrin

Actin

Fig. 3A

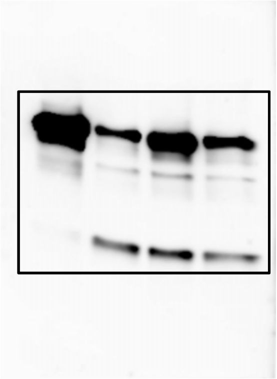

Caspase-3

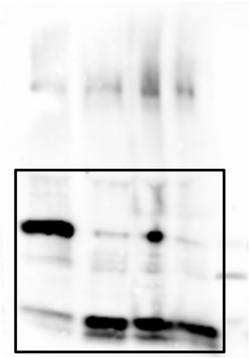

Caspase-7

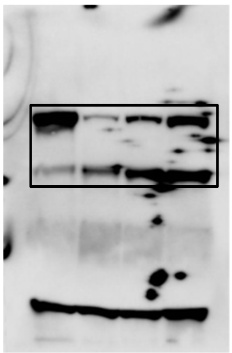

PARP1

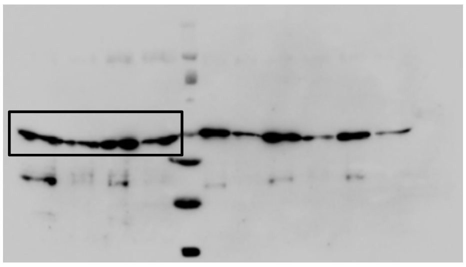

Tubulin

Fig. 3B

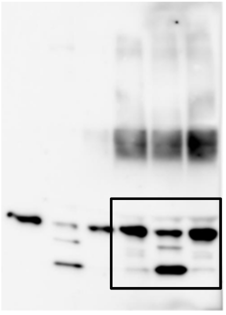

Caspase-3

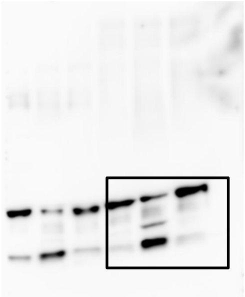

Caspase-7

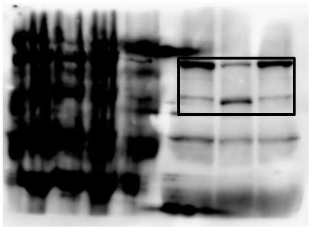

PARP

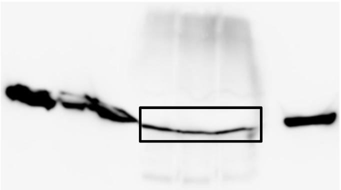

Actin

Fig. 3D

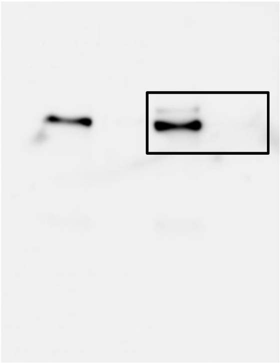

Caspase-3

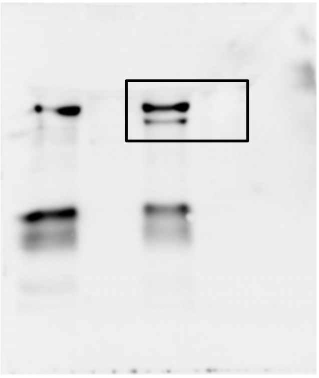

Caspase-7

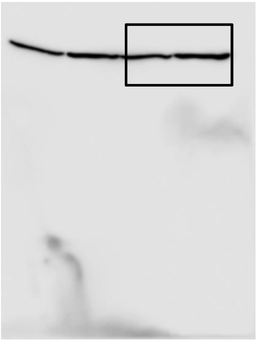

Actin

Fig. 4D

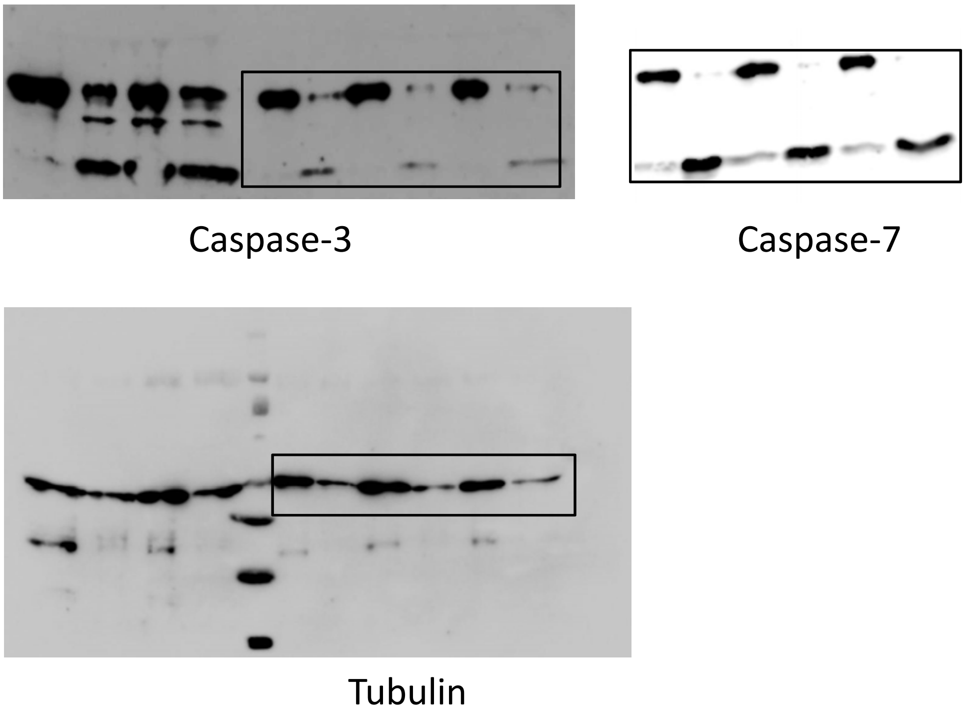

Fig. 4E

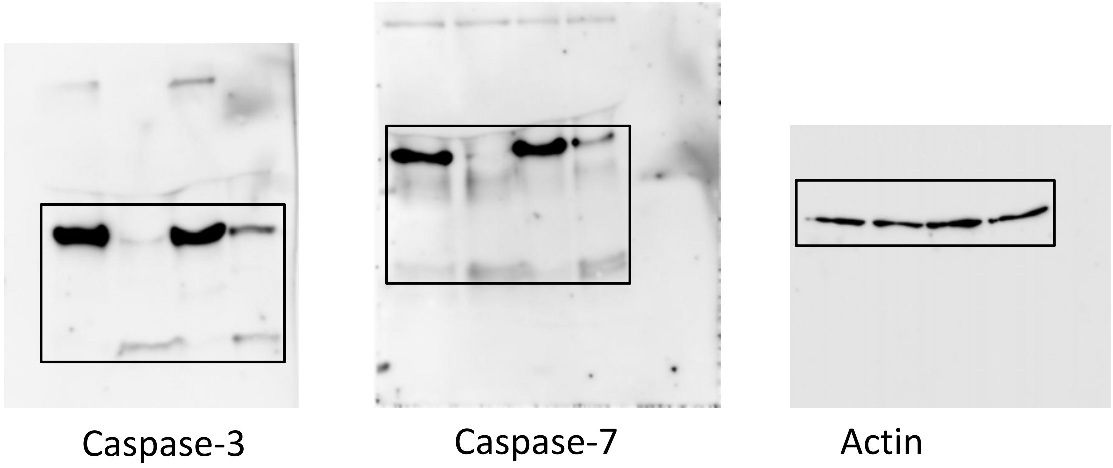

Fig. S4B Fig. S5B

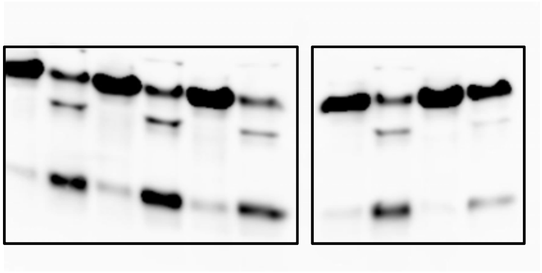

Caspase-3

Fig. S4B Fig. S5B

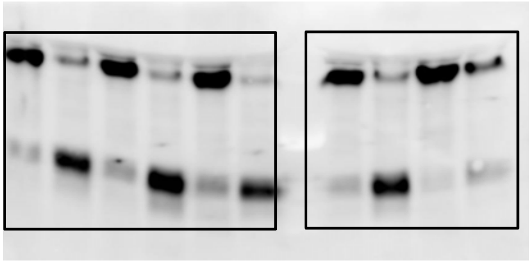

Caspase-7

Fig. S4B Fig. S5B

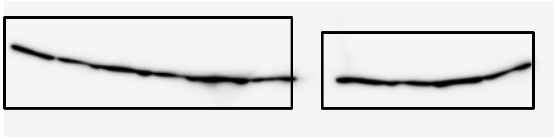

Actin
