# Supplementary material for: Apoptosis of intestinal epithelial cells restricts Clostridium difficile infection in a model of pseudomembranous colitis
Source: Nat Commun. 2018 Nov 19;9:4846. doi: 10.1038/s41467-018-07386-5 (PMC6242954; doi:10.1038/s41467-018-07386-5)
Supplement: Supplementary file 3 — Description of Additional Supplementary Files [file 41467_2018_7386_MOESM3_ESM.pdf]

Description of Additional Supplementary Files

Supplementary Movie 1

Description: *C. difficile* toxins and FlaTox stimulation induce differential cell death morphology in intestinal epithelial cells. Primary intestinal organoids from wildtype mice were stimulated with TcdA, TcdB or FlaTox before PI incorporation was analyzed by time lapse microscopy. The movie shows intervals of 15 minutes per frame during 16 h. Scale bars: 30 µm. Data are representative of 3 independent experiments.
